# Simvastatin impairs fracture healing under ischemic conditions

**DOI:** 10.3389/fphar.2025.1693683

**Published:** 2025-10-15

**Authors:** S. Schreiber, J. Stutz, J. Finkler, W. Metzger, T. Fritz, D. Osche, H. Hawi, S. Razaeian, M. Örgel, M. D. Menger, T. Pohlemann, E. Liodakis, M. W. Laschke, M. Orth

**Affiliations:** ^1^ Department of Trauma, Hand and Reconstructive Surgery, Saarland University, Homburg, Germany; ^2^ Institute for Clinical and Experimental Surgery, Saarland University, PharmaScienceHub (PSH), Homburg, Germany

**Keywords:** simvastatin, ischemia, fracture healing, mouse, BMP-2, CD31, PI3K

## Abstract

Patients suffering from fractures are often required to take simvastatin during the bone healing phase due to co-morbidities. However, the impact of simvastatin on fracture healing under ischemic conditions remains unclear so far. Therefore, we analyzed in this study the effect of simvastatin on fracture healing in an established murine ischemia model. Mild ischemia of the right hind limb and a femoral fracture was induced in CD-1 mice. After stabilization of the fracture by an intramedullary screw, the animals received either 30 mg/kg body weight simvastatin per os daily or an equivalent amount of vehicle (control). Bone healing was analyzed by biomechanical as well as radiological, histomorphometric and Western blot analyses 2 and 5 weeks postoperatively. The fractured femurs of both groups exhibited a delayed healing throughout the study period. Bone formation, as assessed by micro-computed tomography, was significantly reduced in the callus tissue of femurs in simvastatin-treated animals compared to controls. Moreover, these femurs showed histomorphometric signs of ongoing healing and a tendency towards less bone tissue at 2 weeks after surgery. Western blot analyses revealed an increased expression of CD31 and phosphoinositide-3-kinase (PI3K) after simvastatin treatment, whereas the expression of bone morphogenetic protein (BMP)-2 was significantly decreased. In conclusion, these results demonstrate that simvastatin impairs fracture healing under challenging ischemic conditions. This effect is most likely caused by an imbalance of angiogenesis and osteogenesis in the callus tissue. These findings indicate that the use of simvastatin during fracture healing under ischemic conditions warrants careful reconsideration in clinical practice.

## 1 Introduction

Simvastatin is a well-established inhibitor of the enzyme 3-hydroxy-3-methyl-glutaryl-coenzyme A (HMG-CoA) reductase and is primarily used to treat hypercholesterolemia or combined dyslipidemia in clinical practice (Pedersen and Tobert, 2004). In patients with manifest atherosclerotic heart disease or diabetes mellitus, simvastatin has proven to reduce cardiovascular mortality and morbidity (Strandberg et al., 2024).

Beyond its beneficial effect on cardiovascular diseases, simvastatin has been shown to influence the process of bone healing with controversial outcomes so far (Chen et al., 2010; Li et al., 2011; Song et al., 2008; Thunyakitpisal and Chaisuparat, 2004; Tang et al., 2008; Oxlund et al., 2001; Maritz et al., 2001). *In vitro* studies revealed that simvastatin increases the viability and differentiation of osteoblasts (Chen et al., 2010). In addition, simvastatin stimulates the osseous-anabolic signaling pathways of estrogen receptor-α and inhibits osteoclast activity (Li et al., 2011; Song et al., 2008). On the other hand, simvastatin reduces matrix metalloproteinase (MMP)-9 expression, which may affect the viability and functionality of osteoclasts, osteoblasts and osteocytes and lead to impaired recanalization of bone tissue and angiogenesis as part of the fracture healing cascade (Thunyakitpisal and Chaisuparat, 2004; Tang et al., 2008; Khoswanto, 2023).

Controversial effects of simvastatin on bone tissue have also been reported *in vivo*. While simvastatin increases cancellous bone volume as well as the compressive strength of vertebral bodies under physiological conditions in rats (Oxlund et al., 2001), it has also been shown to decrease dose-dependently bone density in these animals (Maritz et al., 2001).

Many patients suffering from fractures receive simvastatin due to cardiovascular co-morbidities. In fact, utilization of statins lately showed a strong increase of 197% between 2008–2009 and 2018–2019 (Matyori et al., 2023). Although efforts have been made to understand the effect of simvastatin on bone healing under physiological conditions, its effect on bone healing under ischemic conditions has not been analyzed so far. Of interest, ischemic conditions are a major risk factor for delayed bone healing and may even lead to non-union formation (Haffner-Luntzer et al., 2019; Lu et al., 2007). Therefore, we aimed to investigate the effect of simvastatin on delayed bone healing *in vivo* under challenging ischemic conditions. To the best of our knowledge, this is the first study to evaluate simvastatin in an ischemic fracture model.

## 2 Materials and methods

### 2.1 Animals

A total of 44 CD-1 mice (22 male and 22 female mice) with a body weight of 37.5 g ± 5.5 g were used. A power calculation was performed using G*Power (version 3.1.9.7, Axel Buchner, Heinrich-Heine-Universität Düsseldorf, Germany). The animals were bred at the Institute for Clinical and Experimental Surgery, Saarland University, Germany, kept at a regular light and dark cycle (12-h (h) day/night rhythm) and had free access to tap water and standard pellet food (Altromin, Lage, Germany). The study was conducted in accordance with the German legislation on protection of animals and the National Institutes of Health (NIH) Guidelines for the Care and Use of Laboratory Animals and was approved by the local authorities (permission number: 35/2020; State Office for Consumer Protection, Saarbrücken, Germany).

### 2.2 Surgical procedure

**FIGURE 1:**
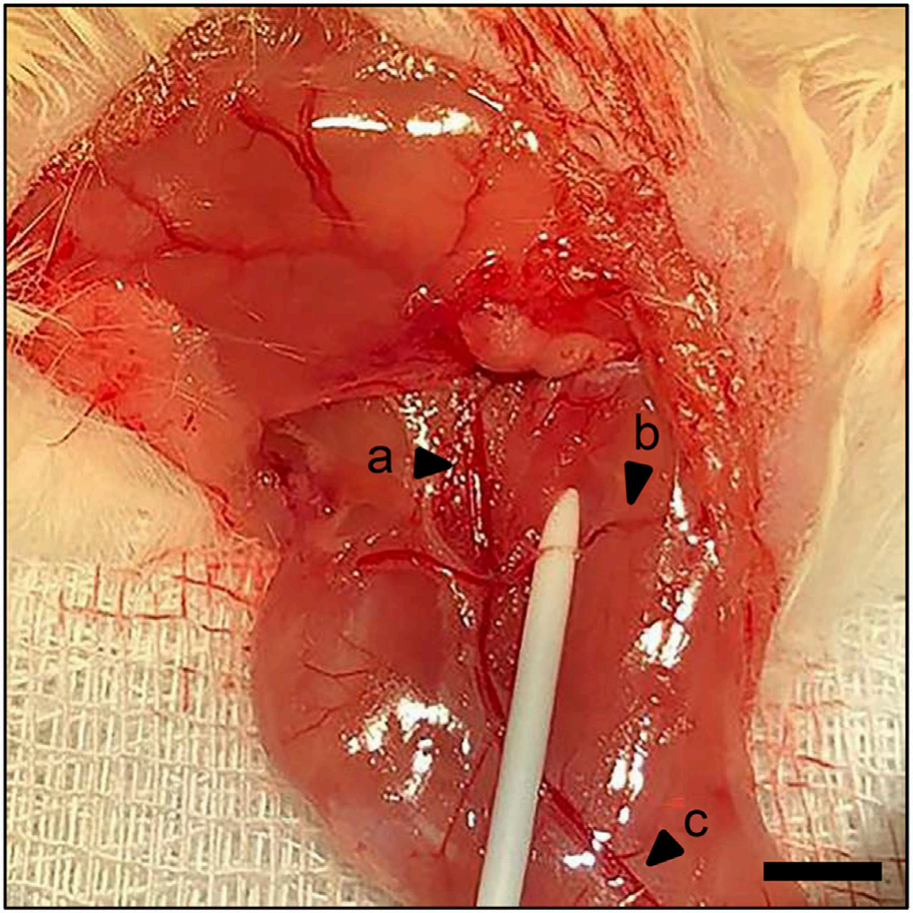
Surgical procedure. Identification of the femoral artery and vein **(a)** and popliteal artery and vein **(c)**. Ischemia is induced by ligation of the deep femoral artery **(b)**. Scale bar: 2000 µm.

The present study used a well-established ischemia model, as previously described in detail (Menger et al., 2022). Briefly, an intraperitoneal administration of 75 mg/kg body weight of ketamine (Pharmacia, Erlangen, Germany) and 12 mg/kg body weight of xylazine 2% (Bayer, Leverkusen, Germany) was used for anesthesia. An 8 mm incision was made on the right hind limb medial to the patella in the direction of the femoral artery parallel to the course of the vessels. The deep femoral artery was ligated twice with a non-absorbable 6–0 suture (Ethicon, Raritan, United States) in order to create ischemic conditions (Figure 1). The knee joint capsule was opened longitudinally along the medial border of the patella, allowing lateral dislocation of the patella to expose the femoral condyles. A hole with a diameter of 0.5 mm was drilled into the intercondylar notch, and then an injection needle with a 0.4 mm diameter was drilled into the intramedullary canal. A tungsten guidewire (0.2 mm in diameter) was inserted through the needle into the intramedullary canal subsequently. After removal of the needle, the femur was fractured using a blunt guillotine with defined weight and height. A titanium intramedullary screw (MouseScrew™, RISystem, Davos, Switzerland) was implanted over the guidewire to stabilize the fracture (Holstein et al., 2009).

Animals of the simvastatin group (n = 22) received 30 mg/kg body weight simvastatin (Hexal AG, Holzkirchen, Germany) daily per os (*via* intragastric gavage) from the day of surgery to achieve a dose comparable to that used in clinical practice (Youssef et al., 2002; Cilla et al., 1996; Dostal et al., 1996).

Animals of the control group (n = 22) received an equivalent volume of the vehicle (NaCl 0.9%; Braun, Melsungen, Germany). Animals were sacrificed by cervical dislocation after 2 weeks (n = 14 control group; n = 13 simvastatin group) or 5 weeks (n = 8 control group; n = 9 simvastatin group) postoperatively. X-ray imaging was performed to confirm the fracture and the implant position. Femurs were harvested and used for further analyses.

### 2.3 Biomechanical analysis

For biomechanical analysis, the right and left femurs were resected at 2 weeks (n = 9 control group; n = 8 simvastatin group) and 5 weeks (n = 8 control group; n = 9 simvastatin group) and freed from soft tissue. After removing the implants, callus stiffness was measured [N/mm] using a three-point bending device (Mini-Zwick Z 2.5; Zwick, Ulm, Germany), as described previously (Orth et al., 2019). Loading was stopped individually in every case when the actual load-displacement curve deviated more than 1% from linearity. The relative bending stiffness [%] was obtained by comparing the results of the fractured femur with the bending stiffness of the healthy contralateral femur. Using this non-destructive approach for biomechanical analyses, the femurs could also be used for subsequent micro-computed tomography (µCT) and histological analyses. This approach resulted in a marked reduction of animals needed, according to the 3R principle (replacement, reduction, refinement) (Díaz et al., 2020).

### 2.4 Radiological analysis

X-rays (MX-20 Faxitron; X-ray Corporation, Wheeling, IL, United States) of the fractured femurs were performed at 2 weeks (n = 9 control group; n = 8 simvastatin group) and 5 weeks (n = 8 control group; n = 9 simvastatin group) after surgery for macroscopic assessment of the injured femurs.

Thereafter, fractured femurs were analyzed by µCT (Skyscan 1176; Bruker, Billerica, United States). For this purpose, the femurs were scanned at a spatial resolution of 7.5 μm with a standardized setup, as described previously (Orth et al., 2019). To express gray values as mineral content (bone mineral density; BMD), calcium hydroxyapatite (CaHA) phantom rods with known BMD values were used for calibration. The region of interest (ROI) was contoured manually on each transversal slide defining exclusively novel bone and excluding original cortical bone. The ROI was processed with a threshold procedure (CTAnalyzer, Bruker), which allowed for differentiation between bone and soft tissue. The thresholds to distinguish between bone and soft tissue were based on visual inspection of the images, qualitative comparison with histological sections and previous studies investigating bone repair and callus tissue by µCT (Orth et al., 2019; Goldberg et al., 1985). A BMD with more than 0.410 g/cm^3^, resulting in gray values of 68–255 was defined as mineralized bone. The following µCT parameters were calculated from the callus ROI for each specimen: ratio of bone volume (BV) to total volume (TV) of the callus (BV/TV [%]), trabecular number (TbN [1/mm]), trabecular separation (TbSp [mm]) and trabecular thickness (TbTh [mm]).

### 2.5 Histomorphometric analysis

For histomorphometric analysis, bones were fixed in 4% formalin solution (Carl Roth, Karlsruhe, Germany) for 24 h and decalcified in ethylenediaminetetraacetic acid (EDTA) solution for 2 weeks. Dehydration was carried out in an ascending alcohol series. Longitudinal sections with a thickness of 5 µm were stained with Safranin-O after embedding decalcified bones in paraffin (control group: n = 9 at 2 weeks and n = 8 at 5 weeks; simvastatin group: n = 7 at 2 weeks and n = 9 at 5 weeks). In order to perform a quantitative measurement, the histological specimens were digitized (Keyence BioZero BZ8100 fluorescence microscope, Keyence Deutschland, Neu-Isenburg, Germany). At a magnification of ×12.5 structural indices were calculated based on the recommendations, as described previously (Gerstenfeld et al., 2005). For histomorphometric evaluation the following parameters were measured: (i) total periosteal callus area [mm^2^], (ii) bone callus area [mm^2^], (iii) cartilaginous callus area [mm^2^] and (iv) connective tissue callus area [mm^2^]. The total periosteal callus area was defined as all osseous, cartilaginous and fibrous callus tissue outside of the cortices. Each area was marked and calculated using the ImageJ Analysis System (NIH, Bethesda, United States).

### 2.6 Western blot

Protein expression within the callus tissue was determined by Western blot analyses, including the expression of cluster of differentiation 31 (CD31), bone morphogenetic protein (BMP)-2, phosphoinositide-3-kinase (PI3K), vascular endothelial growth factor (VEGF), receptor activator of nuclear factor kappa-Β ligand (RANKL) and osteoprotegerin (OPG). After harvesting the callus at 2 weeks after surgery (n = 5 each group), the material was immediately transferred to liquid nitrogen and then stored at −80 °C. After saving the whole protein fraction, proteins were separated and transferred to membranes by standard protocols and probed using anti-CD31 (1:30, Cell Signaling Technology Europe B.V., Frankfurt am Main), anti-BMP-2 (1:30, R&D Systems, Minneapolis, United States), anti-PI3K (1:30, R&D Systems), anti-VEGF (1:100, Abcam, Cambridge, United Kingdom), anti-RANKL (1:30, Abcam) and anti-OPG (1:30, R&D Systems). All antibodies were incubated overnight at 4 °C and then for 4 h at room temperature. The appropriate peroxidase-conjugated anti-IgG antibodies served as secondary antibodies (Dako Agilent Technologies, California, United States and R&D Systems). Protein expression was visualized by means of luminol-enhanced chemiluminescence after exposure of the membrane to the Intas ECL Chemocam Imager (Intas Science Imaging Instrument GmbH, Göttingen, Germany). To correct for unequal loading, signals were normalized to β-actin signals (Santa Cruz Biotechnology, Heidelberg, Germany).

### 2.7 Statistical analysis

The statistical analyses were performed using the SigmaPlot 13.0 software (Systat Software, Inc., San José, United States). All data are given as means ± standard error of the mean (SEM). Statistical outliers (>2 SEM) were not included in the subsequent data analysis. Data were first tested for normal distribution (Shapiro-Wilk test) and equal variance (Brown-Forsythe test). In case of parametric data, comparisons between two experimental groups were performed by an unpaired Student’s t-test. In case of non-parametric distribution, a Mann-Whitney rank sum test was performed. A p-value <0.05 was considered to indicate significant differences.

## 3 Results

### 3.1 Biomechanical analysis

**FIGURE 2:**
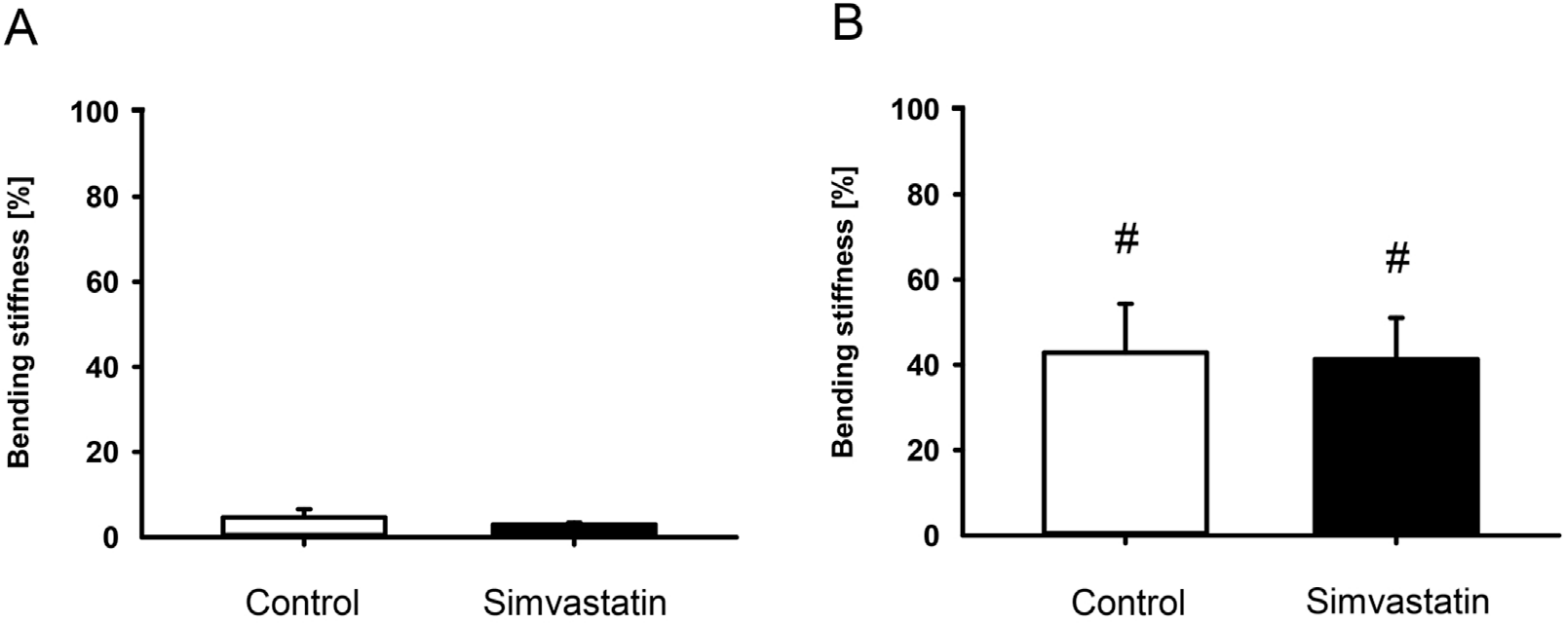
Biomechanical analysis of mouse femurs. **(A,B)** Ratio of bending stiffness of fractured to unfractured control (white; n = 9/8) and simvastatin (black; n = 8/9) femurs at 2 weeks **(A)** and 5 weeks **(B)** after surgery. Mean ± SEM. ^#^p < 0.05 vs. control/simvastatin at 2 weeks.

The absolute bending stiffness in the simvastatin group tended to be slightly lower than in the control group at 2 weeks (control: 5.01 ± 1.46 N/mm; simvastatin: 3.03 ± 0.43 N/mm; p > 0.05) and 5 weeks (control: 51.29 ± 12.68 N/mm; simvastatin: 48.99 ± 12.45 N/mm; p > 0.05) after surgery in the intergroup comparison. This non-significant tendency was also reflected in the relative bending stiffness 2 and 5 weeks after surgery (Figures 2A,B). The intragroup comparison revealed a significant increase of the relative bending stiffness for both groups from 2 to 5 weeks after surgery (Figures 2A,B).

### 3.2 Radiological analysis

**FIGURE 3:**
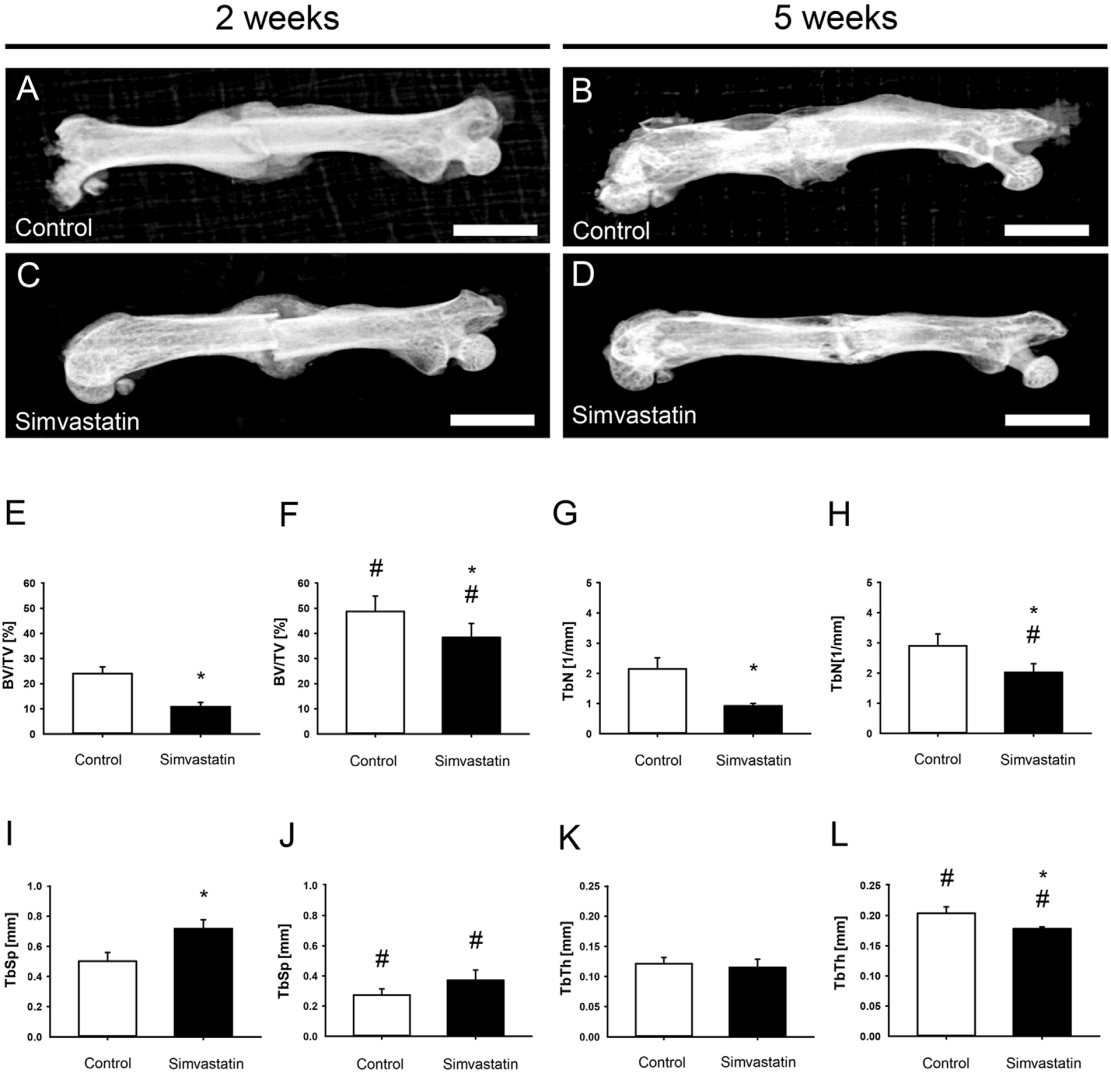
Radiological analysis (X-ray and µCT) of mouse femurs. **(A–D)** X-ray images of femurs at 2 weeks **(A,C)** and 5 weeks **(B,D)** after surgery of control **(A,B)** and simvastatin **(C,D)** animals. Scale bars: 3,000 µm. **(E–L)** Radiological analysis (µCT) of mouse femurs. **(E,F)** Ratio of bone volume to tissue volume (BV/TV) at 2 weeks **(E)** and 5 weeks **(F)** after surgery within the callus of control (white; n = 9/8) and simvastatin (black; n = 8/9) animals. **(G,H)** Trabecular number (TbN) at 2 weeks **(G)** and 5 weeks **(H)** after surgery within the callus of control (white; n = 9/8) and simvastatin (black; n = 8/9) animals. **(I,J)** Trabecular separation (TbSp) at 2 weeks **(I)** and 5 weeks **(J)** after surgery within the callus of control (white; n = 9/8) and simvastatin (black; n = 8/9) animals. **(K,L)** Trabecular thickness (TbTh) at 2 weeks **(K)** and 5 weeks **(L)** after surgery within the callus of control (white; n = 9/8) and simvastatin animals (black; n = 8/9). Mean ± SEM. ^*^p < 0.05 vs. control; ^#^p < 0.05 vs. control/simvastatin at 2 weeks.

The X-rays of fractured femurs demonstrated a radiopaque callus formation with bridging of the fracture site at 2 weeks in both groups (Figures 3A,C). At 5 weeks after surgery, the callus exhibited signs of remodeling (Figures 3B,D). Of interest, µCT analyses revealed a significantly reduced absolute bone volume in the callus of simvastatin-treated animals compared to controls after 2 weeks (control: 6.41 ± 0.92 mm^3^; simvastatin: 3.30 ± 0.37 mm^3^; p < 0.05) and after 5 weeks (control: 8.75 ± 1.48 mm^3^; simvastatin: 4.08 ± 0.89 mm^3^; p < 0.05). Accordingly, BV/TV was also significantly reduced after treatment of the animals with simvastatin compared to control animals at 2 and 5 weeks (Figures 3E,F). The intragroup comparison of BV/TV between results at 2 and 5 weeks showed a significant increase of BV/TV in both groups. Trabecular parameters of the µCT analysis revealed a significantly reduced TbN at 2 and 5 weeks in animals of the simvastatin group (Figures 3G,H). Furthermore, TbSp was increased at 2 weeks and TbTh was reduced at 5 weeks in femurs of the simvastatin group (Figures 3I–L).

### 3.3 Histomorphometric analysis

**FIGURE 4:**
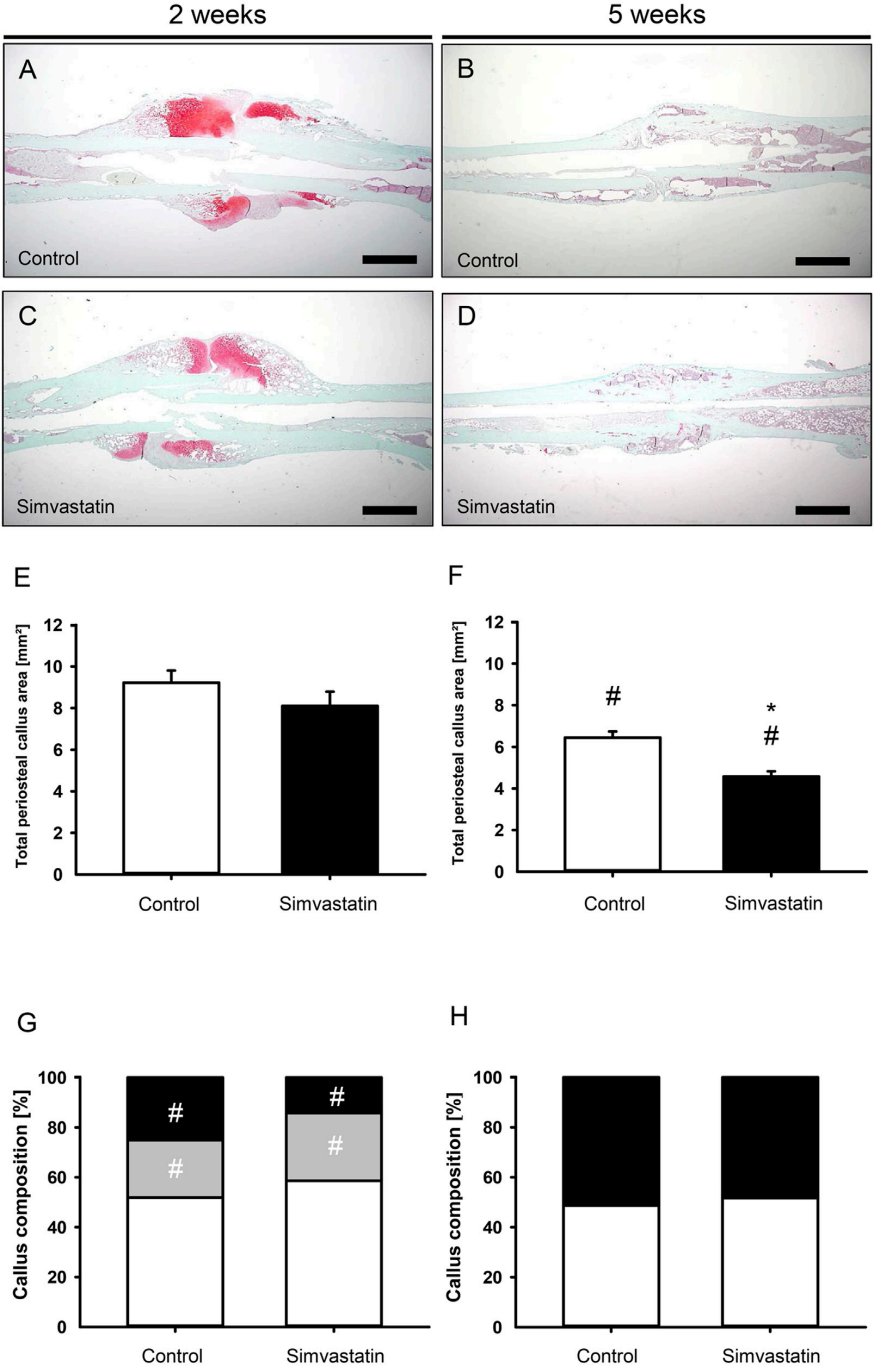
Histomorphometric analysis of mouse femurs. **(A–D)** Representative histological images of Safranin-O-stained femurs at 2 weeks **(A,C)** and 5 weeks **(B,D)** after surgery of control **(A,B)** and simvastatin **(C,D)** animals. Scale bars: 1000 µm. **(E,F)** Total periosteal callus area at 2 weeks **(E)** and 5 weeks **(F)** after surgery of control (white; n = 9/8) and simvastatin (black; n = 7/9) animals. **(G,H)** Callus composition. Fraction of osseous callus (black), cartilaginous callus (gray) and connective tissue (white) of the total callus area of control (left column; n = 9/8) and simvastatin (right column; n = 7/9) animals at 2 weeks **(G)** and 5 weeks **(H)** after surgery. Mean ± SEM; ^*^p < 0.05 vs. control; ^#^p < 0.05 vs. control/simvastatin at 2/5 weeks.

Histomorphometric analyses of the fractured femurs 2 weeks after surgery showed callus formation at the site of injury that was lacking osseous bridging of the fracture in both groups at this early time point (Figures 4A,C) compared to femurs at 5 weeks after surgery (Figures 4B, D). Five weeks postoperatively, the total periosteal callus area in the simvastatin group was significantly reduced compared to the controls (control: 6.45 ± 0.29 mm^2^; simvastatin: 4.58 ± 0.25 mm^2^; *p* < 0.05) (Figures 4E, F). Analyses of the callus composition revealed a tendency of less bone in animals of the simvastatin group at 2 weeks after surgery, however, without proven to be significantly different (2 weeks: control: 25.12% ± 5.26%; simvastatin: 14.22% ± 2.98%; 5 weeks: control: 50.19% ± 4.62%; simvastatin: 47.27% ± 7.13%). The proportion of bone increased significantly throughout the study period in both groups (Figures 4G, H). In contrast, the intragroup comparison of cartilaginous tissue showed a significant decrease over time with very little cartilage at 5 weeks after surgery in both groups without significant differences in the intergroup comparison (Figures 4G, H). The analysis of connective tissue did not show any differences between the groups (Figures 4G, H).

### 3.4 Western blot analysis

**FIGURE 5:**
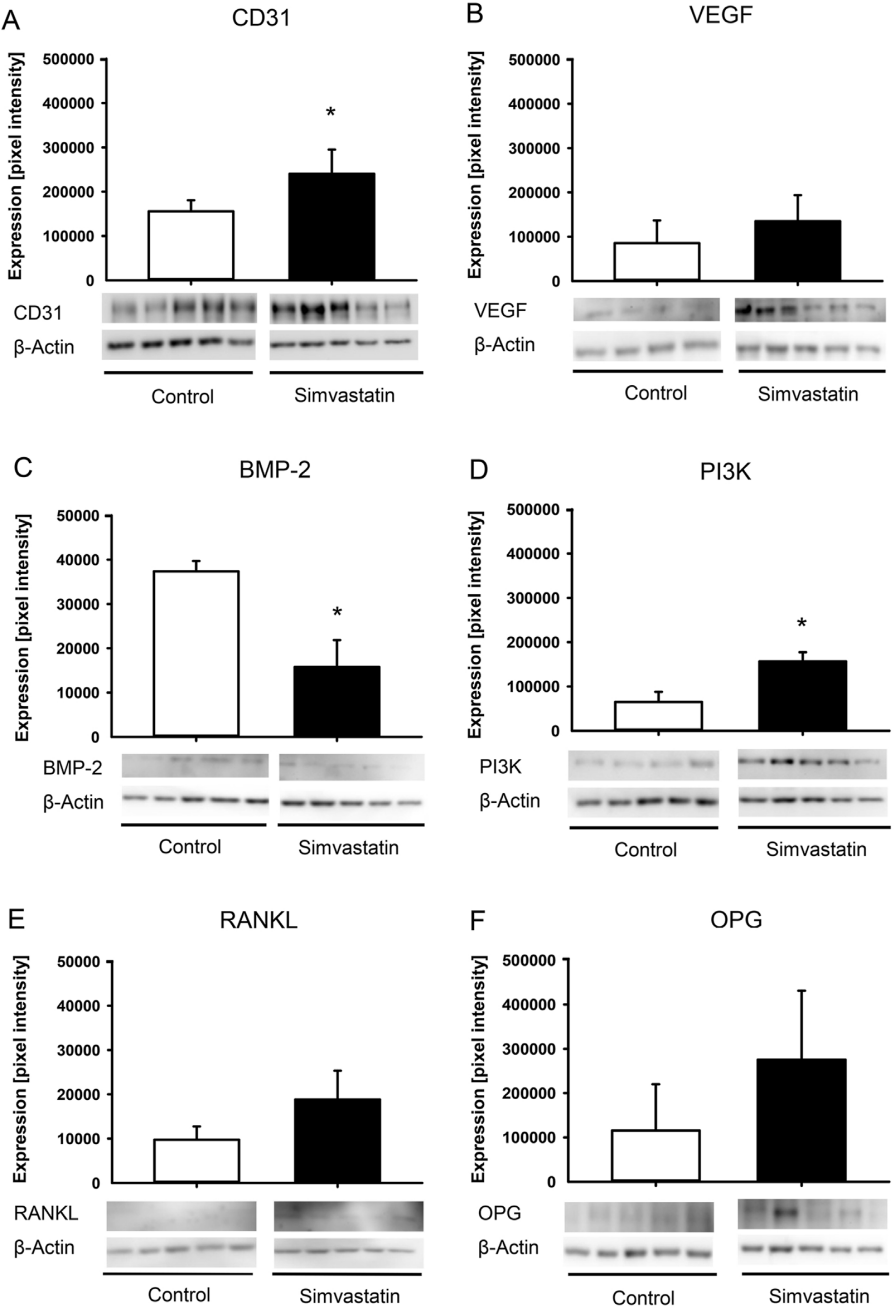
Western blot analysis of callus tissue. **(A–F)** Western blots and expression of CD31 **(A)**, VEGF **(B)**, BMP-2 **(C)**, PI3K **(D)**, RANKL **(E)**, OPG **(F)** and β-actin within the callus tissue of control (white; n = 5) and simvastatin (black; n = 5) femurs at 2 weeks after surgery. Mean ± SEM; ^*^p < 0.05 vs. control.

The expression of the blood vessel marker CD31 was significantly higher in the callus of fractured femurs in simvastatin-treated animals (Figure 5A). Moreover, VEGF was found to be slightly increased, however, without significant differences (Figure 5B). Of interest, the osteogenic marker BMP-2 was observed to exhibit significantly lower expression in the callus tissue of femurs in the simvastatin group when compared to controls (Figure 5C). The amount of PI3K was significantly higher in simvastatin-treated animals compared to controls (Figure 5D). In the context of osteoclastogenesis, the levels of RANKL and OPG tended to be higher in simvastatin-treated animals than in control animals, however, without significant differences (Figures 5E,F).

## 4 Discussion

This study demonstrates for the first time that simvastatin impairs fracture healing under ischemic conditions in a well-established murine model of delayed fracture healing. This effect was mainly observed at the early time point of 2 weeks after surgery.

The pharmacological effect of simvastatin is mediated by the inhibition of the enzyme HMG-CoA reductase, which results in a reduction of cholesterol synthesis (Pedersen and Tobert, 2004; Strandberg et al., 2024). In addition to several pleiotropic effects, simvastatin has been proven to influence bone metabolism under physiological conditions with controversial results in the recent literature (Chen et al., 2010; Li et al., 2011; Song et al., 2008; Thunyakitpisal and Chaisuparat, 2004; Tang et al., 2008; Oxlund et al., 2001; Maritz et al., 2001; Du et al., 2009; Papadimitriou et al., 2015; Skoglund et al., 2002; Skoglund and Aspenberg, 2007).

Simvastatin has been shown to stimulate the bone-anabolic signaling pathways of estrogen receptor-α, which results in a reduced expression of RANKL and increased expression of OPG, thereby inhibiting osteoclast activity (Li et al., 2011; Song et al., 2008; Ghosh-Choudhury et al., 2007). The Western blot analyses showed no significant differences in RANKL/OPG expression. Moreover, the expression of the osteogenic marker BMP-2 was significantly reduced. Hence, it may be speculated that the ischemic conditions in the used model may have markedly compromised the effect of simvastatin on bone healing compared to physiological conditions by reducing its effect on the RANKL/OPG pathway and disturbing its osteogenic activity.

Of interest, simvastatin has been reported to have detrimental effects on bone metabolism by downregulating the activity of MMP-9 (Thunyakitpisal and Chaisuparat, 2004; Tang et al., 2008; Khoswanto, 2023), which may therefore disturb the bone healing process (Thunyakitpisal and Chaisuparat, 2004; Tang et al., 2008; Khoswanto, 2023). As a limitation, the expression of MMP-9 was not investigated in the present study. Further studies will be helpful to analyze, whether the detrimental effect of simvastatin on bone healing under ischemic conditions observed in the present study was caused by affecting the MMP-9 pathway.

The controversial effects of simvastatin on bone metabolism and fracture healing reported in the current literature may also be explained by the use of different doses, species, cell lines and statin types (Li et al., 2011; Maritz et al., 2001; Staal et al., 2003; von Stechow et al., 2003; Chissas et al., 2010). Chissas et al. investigated the effect of simvastatin at two different oral doses on fracture healing after ulnar osteotomy in rabbits (Chissas et al., 2010). Doses of 10 mg/kg body weight per day showed neither a positive nor a negative effect on bone healing. However, at a dose of 30 mg/kg body weight, equivalent to the dosage used in this study, administration of simvastatin resulted in a reduced bone mineral density, bending stiffness and callus formation (Chissas et al., 2010). The authors assumed that osteoblasts and osteoclasts differ in their sensitivity to certain substances (Chissas et al., 2010). Accordingly, it is possible that osteoclasts are sensitive to simvastatin at a higher dose leading to increased bone resorption processes. In line with these results, the reduced BV, BV/TV and trabecular parameters in µCT analysis as well as the negative histomorphometric results in the simvastatin-treated animals of the present study may have been caused by a dose-dependent effect. These results may also be relevant in a clinical context, given that the chosen dosage is comparable to that used in clinical practice (Youssef et al., 2002; Cilla et al., 1996; Dostal et al., 1996). However, this detrimental effect does not appear to cause a non-union at the fracture site, as observed by an increase of bone tissue and signs of remodeling at 5 weeks after surgery compared to results at 2 weeks in simvastatin-treated animals. In fact, most of the biomechanical, radiological and histomorphometric results at 5 weeks in the simvastatin group were similar to animals of the control group and, thus, demonstrated a typical course of delayed bone healing, as described previously (Menger et al., 2022).

The detrimental effect of simvastatin appeared to be most prominent at the early time point of 2 weeks after surgery. Of interest, Lima et al. (2011) and Calixto et al. (2011) observed a strong local inflammatory reaction involving edema, necrosis and encrustation in some cases of high-dose local administration of simvastatin. It may be speculated that the increased expression of PI3K, which is known to regulate several key processes in the inflammatory response to injury and infection (Hawkins and Stephens, 2015), is due to an altered inflammatory response in the early phase after commencement of the simvastatin therapy (Marsell and Einhorn, 2011).

It is well-known that during the early phase of bone healing, angiogenesis takes place and osteogenesis follows neovascularization (Carano and Filvaroff, 2003). The interaction of endothelial cells and osteoblasts are of pivotal importance for a successful healing throughout this highly orchestrated process (Carano and Filvaroff, 2003). In this study, expression of angiogenic CD31 was significantly higher at 2 weeks after surgery in simvastatin-treated animals. In contrast, the expression of osteogenic BMP-2 was significantly reduced at this time point. This resulted in a significantly altered ratio of angiogenic to osteogenic markers within the callus tissue. The essential role of this ratio in fracture healing has been described previously (Orth et al., 2018; Orth et al., 2022). In fact, Orth et al. reported that excessive stimulation of angiogenesis can impair bone formation and fracture healing under physiological conditions (Orth et al., 2018). In contrast, controlled administration of growth factors with a defined pro-osteogenic ratio of angiogenesis/osteogenesis (VEGF:BMP-2 (1:2)) has been reported to improve bone healing and to prevent non-union formation (Orth et al., 2022). Although sufficient angiogenesis is essential for fracture healing to deliver nutrients, the administration of simvastatin may have shifted this highly important ratio of angiogenic and osteogenic markers towards angiogenesis at the fracture site.

In conclusion, these novel findings demonstrate that simvastatin impairs early fracture healing under ischemic conditions in a challenging murine model, however, without completely preventing bone healing. This effect is most likely due to a pro-angiogenic shift in the ratio of angiogenic and osteogenic expression markers in the callus tissue at an early phase of the healing process. Based on these results, simvastatin treatment of fracture patients suffering from tissue ischemia may not be recommended.

## Data Availability

The original contributions presented in the study are included in the article/supplementary material, further inquiries can be directed to the corresponding author.

## References

[B1] Calixto J. C. Lima C. E. Frederico L. Lima R. P. Anbinder A. L. (2011). The influence of local administration of simvastatin in calvarial bone healing in rats. J. Craniomaxillofac Surg. 39 (3), 215–220. 10.1016/j.jcms.2010.03.009 20456964

[B2] Carano R. A. Filvaroff E. H. (2003). Angiogenesis and bone repair. Drug Discov. Today 8 (21), 980–989. 10.1016/s1359-6446(03)02866-6 14643161

[B3] Chen P. Y. Sun J. S. Tsuang Y. H. Chen M. H. Weng P. W. Lin F. H. (2010). Simvastatin promotes osteoblast viability and differentiation *via* Ras/Smad/Erk/BMP-2 signaling pathway. Nutr. Res. 30 (3), 191–199. 10.1016/j.nutres.2010.03.004 20417880

[B4] Chissas D. Stamatopoulos G. Verettas D. Kazakos K. Papalois A. Agrogiannis G. (2010). Can low doses of simvastatin enhance fracture healing? An experimental study in rabbits. Injury 41 (7), 687–692. 10.1016/j.injury.2009.10.011 19880111

[B5] Cilla D. D. Jr Whitfield L. R. Gibson D. M. Sedman A. J. Posvar E. L. (1996). Multiple-dose pharmacokinetics, pharmacodynamics, and safety of atorvastatin, an inhibitor of HMG-CoA reductase, in healthy subjects. Clin. Pharmacol. Ther. 60 (6), 687–695. 10.1016/S0009-9236(96)90218-0 8988072

[B6] Díaz L. Zambrano E. Flores M. E. Contreras M. Crispín J. C. Alemán G. (2020). Ethical considerations in animal research: the principle of 3R's. Rev. Invest Clin. 73 (4), 199–209. Published 2020 May 7. 10.24875/RIC.20000380 33090120

[B7] Dostal L. A. Whitfield L. R. Anderson J. A. (1996). Fertility and general reproduction studies in rats with the HMG-CoA reductase inhibitor, atorvastatin. Fundam. Appl. Toxicol. 32 (2), 285–292. 10.1006/faat.1996.0132 8921332

[B8] Du Z. Chen J. Yan F. Xiao Y. (2009). Effects of simvastatin on bone healing around titanium implants in osteoporotic rats. Clin. Oral Implants Res. 20 (2), 145–150. 10.1111/j.1600-0501.2008.01630.x 19077150

[B9] Gerstenfeld L. C. Wronski T. J. Hollinger J. O. Einhorn T. A. (2005). Application of histomorphometric methods to the study of bone repair. J. Bone Min. Res. 20 (10), 1715–1722. 10.1359/JBMR.050702 16160729

[B10] Ghosh-Choudhury N. Mandal C. C. Choudhury G. G. (2007). Statin-induced ras activation integrates the phosphatidylinositol 3-kinase signal to akt and MAPK for bone morphogenetic protein-2 expression in osteoblast differentiation. J. Biol. Chem. 282 (7), 4983–4993. 10.1074/jbc.M606706200 17179158

[B11] Goldberg V. M. Powell A. Shaffer J. W. Zika J. Bos G. D. Heiple K. G. (1985). Bone grafting: role of histocompatibility in transplantation. J. Orthop. Res. 3 (4), 389–404. 10.1002/jor.1100030401 3906062

[B12] Haffner-Luntzer M. Hankenson K. D. Ignatius A. Pfeifer R. Khader B. A. Hildebrand F. (2019). Review of animal models of comorbidities in fracture-healing research. J. Orthop. Res. 37 (12), 2491–2498. 10.1002/jor.24454 31444806

[B13] Hawkins P. T. Stephens L. R. (2015). PI3K signalling in inflammation. Biochim. Biophys. Acta 1851 (6), 882–897. 10.1016/j.bbalip.2014.12.006 25514767

[B14] Holstein J. H. Matthys R. Histing T. Becker S. C. Fiedler M. Garcia P. (2009). Development of a stable closed femoral fracture model in mice. J. Surg. Res. 153 (1), 71–75. 10.1016/j.jss.2008.02.042 18656902

[B15] Khoswanto C. (2023). Role of matrix metalloproteinases in bone regeneration: narrative review. J. Oral Biol. Craniofac Res. 13 (5), 539–543. 10.1016/j.jobcr.2023.06.002 37351418 PMC10282173

[B16] Li X. Song Q. S. Wang J. Y. Leng H. J. Chen Z. Q. Liu Z. J. (2011). Simvastatin induces estrogen receptor-alpha expression in bone, restores bone loss, and decreases ERα expression and uterine wet weight in ovariectomized rats. J. Bone Min. Metab. 29 (4), 396–403. 10.1007/s00774-010-0231-y 21063740

[B17] Lima C. E. Calixto J. C. Anbinder A. L. (2011). Influence of the association between simvastatin and demineralized bovine bone matrix on bone repair in rats. Braz Oral Res. 25 (1), 42–48. 10.1590/s1806-83242011000100008 21359450

[B18] Lu C. Miclau T. Hu D. Marcucio R. S. (2007). Ischemia leads to delayed union during fracture healing: a mouse model. J. Orthop. Res. 25 (1), 51–61. 10.1002/jor.20264 17019699 PMC2848995

[B19] Maritz F. J. Conradie M. M. Hulley P. A. Gopal R. Hough S. (2001). Effect of statins on bone mineral density and bone histomorphometry in rodents. Arterioscler. Thromb. Vasc. Biol. 21 (10), 1636–1641. 10.1161/hq1001.097781 11597938

[B20] Marsell R. Einhorn T. A. (2011). The biology of fracture healing. Injury 42 (6), 551–555. 10.1016/j.injury.2011.03.031 21489527 PMC3105171

[B21] Matyori A. Brown C. P. Ali A. Sherbeny F. (2023). Statins utilization trends and expenditures in the U.S. before and after the implementation of the 2013 ACC/AHA guidelines. Saudi Pharm. J. 31 (6), 795–800. 10.1016/j.jsps.2023.04.002 37228328 PMC10203693

[B22] Menger M. M. Stutz J. Ehnert S. Nussler A. K. Rollmann M. F. Herath S. C. (2022). Development of an ischemic fracture healing model in mice. Acta Orthop. 93, 466–471. 10.2340/17453674.2022.2529 35478260 PMC9047454

[B25] Orth M. Altmeyer M. A. B. Scheuer C. Braun B. J. Holstein J. H. Eglin D. (2018). Effects of locally applied adipose tissue-derived microvascular fragments by thermoresponsive hydrogel on bone healing. Acta Biomater. 77, 201–211. 10.1016/j.actbio.2018.07.029 30030175

[B26] Orth M. Baudach J. Scheuer C. Osche D. Veith N. T. Braun B. J. (2019). Erythropoietin does not improve fracture healing in aged mice. Exp. Gerontol. 122, 1–9. 10.1016/j.exger.2019.04.005 30998964

[B27] Orth M. Fritz T. Stutz J. Scheuer C. Ganse B. Bullinger Y. (2022). Local application of mineral-coated microparticles loaded with VEGF and BMP-2 induces the healing of murine atrophic non-unions. Front. Bioeng. Biotechnol. 9, 809397. 10.3389/fbioe.2021.809397 35087807 PMC8787303

[B28] Oxlund H. Dalstra M. Andreassen T. T. (2001). Statin given perorally to adult rats increases cancellous bone mass and compressive strength. Calcif. Tissue Int. 69 (5), 299–304. 10.1007/s00223-001-2027-5 11768201

[B29] Papadimitriou K. Karkavelas G. Vouros I. Kessopoulou E. Konstantinidis A. (2015). Effects of local application of simvastatin on bone regeneration in femoral bone defects in rabbit. J. Craniomaxillofac Surg. 43 (2), 232–237. 10.1016/j.jcms.2014.11.011 25534041

[B30] Pedersen T. R. Tobert J. A. (2004). Simvastatin: a review. Expert Opin. Pharmacother. 5 (12), 2583–2596. 10.1517/14656566.5.12.2583 15571475

[B32] Skoglund B. Aspenberg P. (2007). Locally applied simvastatin improves fracture healing in mice. BMC Musculoskelet. Disord. 8, 98. 10.1186/1471-2474-8-98 17897477 PMC2200653

[B33] Skoglund B. Forslund C. Aspenberg P. (2002). Simvastatin improves fracture healing in mice. J. Bone Min. Res. 17 (11), 2004–2008. 10.1359/jbmr.2002.17.11.2004 12412808

[B34] Song C. Wang J. Song Q. Li X. Chen Z. Ma Q. (2008). Simvastatin induces estrogen receptor-alpha (ER-alpha) in murine bone marrow stromal cells. J. Bone Min. Metab. 26 (3), 213–217. 10.1007/s00774-007-0820-6 18470660

[B35] Staal A. Frith J. C. French M. H. Swartz J. Güngör T. Harrity T. W. (2003). The ability of statins to inhibit bone resorption is directly related to their inhibitory effect on HMG-CoA reductase activity. J. Bone Min. Res. 18 (1), 88–96. 10.1359/jbmr.2003.18.1.88 12510809

[B36] Strandberg T. E. Kovanen P. T. Lloyd-Jones D. M. Raal F. J. Santos R. D. Watts G. F. (2024). Drugs for dyslipidaemia: the legacy effect of the Scandinavian simvastatin survival study (4S). Lancet 404 (10470), 2462–2475. 10.1016/S0140-6736(24)02089-0 39577453

[B37] Tang Q. O. Tran G. T. Gamie Z. Graham S. Tsialogiannis E. Tsiridis E. (2008). Statins: under investigation for increasing bone mineral density and augmenting fracture healing. Expert Opin. Investig. Drugs 17 (10), 1435–1463. 10.1517/13543784.17.10.1435 18808306

[B38] Thunyakitpisal P. D. Chaisuparat R. (2004). Simvastatin, an HMG-CoA reductase inhibitor, reduced the expression of matrix metalloproteinase-9 (gelatinase B) in osteoblastic cells and HT1080 fibrosarcoma cells. J. Pharmacol. Sci. 94 (4), 403–409. 10.1254/jphs.94.403 15107580

[B39] Von Stechow D. Fish S. Yahalom D. Bab I. Chorev M. Müller R. (2003). Does simvastatin stimulate bone formation *in vivo?* BMC Musculoskelet. Disord. 4, 8. 10.1186/1471-2474-4-8 12718758 PMC156891

[B40] Youssef S. Stüve O. Patarroyo J. C. Ruiz P. J. Radosevich J. L. Hur E. M. (2002). The HMG-CoA reductase inhibitor, atorvastatin, promotes a Th2 bias and reverses paralysis in central nervous system autoimmune disease. Nature 420 (6911), 78–84. 10.1038/nature01158 12422218

